# Breast cancer risk in male twins: joint analyses of four twin cohorts in Denmark, Finland, Sweden and the United States

**DOI:** 10.1054/bjoc.2000.1468

**Published:** 2000-11

**Authors:** D C Whiteman, M F G Murphy, P K Verkasalo, W F Page, B Floderus, A Skytthe, N V Holm

**Affiliations:** ICRF General Practice Research Group, ICRF Cancer Epidemiology Unit, University of Oxford, Oxford, UK; Department of Public Health, University of Helsinki, Helsinki, Finland; Medical Follow-up Agency, Institute of Medicine, National Academy of Sciences, Washington, DC, USA; Institute of Environmental Medicine, Karolinska Institute, Stockholm, Sweden; Danish Twin Registry & Epidemiology, Institute of Public Health; Danish Centre for Demographic Research, University of Southern Denmark, Odense, Denmark

**Keywords:** breast cancer, twins, oestrogen, pregnancy, risk factors

## Abstract

To test the hypothesis that in utero exposure to high levels of oestrogen increases the risk of male breast cancer, we followed 115 235 male twins for more than 3.5 million person-years at risk. We observed 11 cases of male breast cancer versus 16.16 expected based on national rates (standardized rate ratio 0.68, 95% confidence interval 0.34–1.22) and conclude that any adverse influence of in utero oestrogen exposure is likely to be small. © 2000 Cancer Research Campaign


*British Journal of Cancer* (2000) **83**(9), 1231–1233
© 2000 Cancer Research Campaign
doi: 10.1054/ bjoc.2000.1468, available online at http://www.idealibrary.com on IDEAL®

Short Communication

### Breast cancer risk in male twins: joint analyses of four twin cohorts in Denmark, Finland, Sweden and the United States

**DC Whiteman[1], MFG Murphy[1], PK Verkasalo[2,3], WF Page[4], B Floderus[5], A Skytthe[6,7] and NV Holm[6]**

[1]ICRF General Practice Research Group & [2]ICRF Cancer Epidemiology Unit, University of Oxford, Oxford, UK; [3]Department of Public Health, University of Helsinki, Helsinki, Finland; [4]Medical Follow-up Agency, Institute of Medicine, National Academy of Sciences, Washington DC, USA; [5]Institute of Environmental Medicine, Karolinska Institute, Stockholm, Sweden; [6]Danish Twin Registry & Epidemiology, Institute of Public Health, [7]Danish Centre for Demographic Research, University of Southern Denmark, Odense, Denmark

**Summary** To test the hypothesis that in utero exposure to high levels of oestrogen increases the risk of male breast cancer, we followed 115 235 male twins for more than 3.5 million person-years at risk. We observed 11 cases of male breast cancer versus 16.16 expected based on national rates (standardized rate ratio 0.68, 95% confidence interval 0.34–1.22) and conclude that any adverse influence of in utero oestrogen exposure is likely to be small. © 2000 Cancer Research Campaign

**Keywords:** breast cancer; twins; oestrogen; pregnancy; risk factors

In utero fetal exposure to high oestrogen levels has been proposed as a causal factor in the aetiology of female breast cancer (Trichopoulos, 1990). Isolating this effect in females is complicated by a variety of postnatal hormonal exposures including menarche, menstrual cycles, pregnancies, menopause and exogenous hormones. Since breast cancer in males is considered to be essentially the same disease as in females (based on similarities in histopathology (Thomas, 1993), genetics (Friedman et al, 1997; Mavraki et al, 1997) and other predisposing factors (Sasco et al, 1993)), any increased risk from exposure to high levels of oestrogen in utero should be easier to detect in males due to the absence of these complicating postnatal factors. During gestation, all twins are exposed to levels of unbound endogenous oestrogen that are approximately two-fold greater than for singletons (TambyRaja and Ratnam, 1981; Thomas et al, 1998), and should therefore be at higher risk for male breast cancer assuming the 'in utero exposure hypothesis' to be true (London, 1993). We have analysed the occurrence of male breast cancer (MBC) in 115 235 male twins followed for more than 3.5 million person-years at risk to test the hypothesis that prenatal exposure to high levels of oestrogen increases the risk of MBC.

Male-male twin pairs were identified from population-based twin registers in three Nordic countries (Denmark, Finland, Sweden) as well as a large US twin register (the National Academy of Science – National Research Council Twin registry). The Danish cohort consisted of 8970 twin individuals born between 1870–1930 who were both alive on January 1, 1943 (Hauge, 1981). The Finnish twin cohort included 15 813 male twins who were born between 1880–1958 and were both alive and living in Finland on December 31, 1975 (Verkasalo et al, 1999a).

*Received 29 February 2000*
*Revised 3 August 2000*
*Accepted 4 August 2000*

*Correspondence to:* Dr David Whiteman

We also analysed two sub-cohorts of the Swedish twin registry: an older cohort comprising 9680 male twins born between 1886 and 1925 who were both alive in 1961 (Cederlof et al, 1977) and a younger cohort of 11964 male twins of known zygosity born between 1926 and 1958 who were both alive in 1971, and 11826 twins of unknown zygosity born 1926–67 (Medlund et al, 1976). For the Swedish twin registry only, we were also able to ascertain a further cohort of 13 820 male co-twins from unlike-sex twins born 1926–1967. In each of these Nordic cohorts, zygosity was determined by self-reported questionnaire items and incident cases of MBC (both fatal and non-fatal) were identified through record linkage to the national cancer registry in each country for which ascertainment is virtually complete (Lichtenstein et al, 2000).

The US twin cohort was derived from the National Academy of Science – National Research Council Twin registry (Jablon et al, 1967), and comprised 31 848 white male twins who were born in the years 1917–1927 inclusive and served in the US military during World War 2. Unlike the Nordic cohorts, this historical cohort was followed up only for mortality using cause of death certificates from the Department of Veterans Affairs and the National Death Index files.

For all four cohorts, observed numbers of cancers and person-years at risk were calculated according to age (five-year age-groups), calendar period (varied by country) and zygosity (monozygous (MZ), dizygous (DZ) and unknown zygosity (UZ)). We stratified the analyses by zygosity firstly to determine whether the risk of MBC differed between MZ and DZ twins, and secondly because we were concerned that the follow-up information for those coded as 'unknown zygosity' may have differed qualitatively from those of known zygosity.

Calculation of person-years began at compilation of the cohort and ended at death or closure of follow-up (Denmark: 1943–93; Finland: 1976–95; Sweden: 1961 (older cohort)/1971 (younger cohort)–1996; US: 1946–95). The expected numbers were calculated by multiplying the stratum-specific numbers of person-years

**Table 1** Occurrence of breast cancer among male twins, by country

| Country | No of twin individuals | PYAR[a] | Obs[b] | Exp[c] | SRR[d] | 95% CI[e] |
|---|---|---|---|---|---|---|
| Denmark 1943–93 (born 1870–1930) | | | | | | |
| MZ[f] | 2808 | 105 221 | 1 | 1.30 | 0.77 | 0.02–4.29 |
| DZ | 5256 | 198 688 | 3 | 2.25 | 1.33 | 0.28–3.90 |
| UZ | 906 | 28 877 | 0 | 0.33 | 0 | 0–11.2 |
| Total | 8970 | 332 786 | 4 | 3.88 | 1.03 | 0.39–2.75 |
| Finland 1976–95 (born < 1958) | | | | | | |
| MZ | 3770 | 68 817 | 0 | 0.31 | 0 | 0–11.9 |
| DZ | 8936 | 163 979 | 0 | 0.70 | 0 | 0–5.27 |
| UZ | 3107 | 52 874 | 2 | 0.28 | 7.16 | 0.87–25.9 |
| Total | 15 813 | 285 670 | 2 | 1.29 | 1.55 | 0.19–5.60 |
| Sweden 1961/71–96 Older cohort (born 1886–1925) | | | | | | |
| MZ | 3298 | 83 804 | 1 | 1.55 | 0.64 | 0.02–3.59 |
| DZ | 5966 | 153 938 | 2 | 2.72 | 0.74 | 0.09–2.66 |
| UZ | 416 | 10 410 | 0 | 0.19 | 0 | 0–19.4 |
| Total | 9680 | 248 152 | 3 | 4.46 | 0.67 | 0.14–1.97 |
| Younger cohort (born 1926–58) | | | | | | |
| MZ | 4584 | 112 268 | 0 | 0.33 | 0 | 0–11.2 |
| DZ | 7380 | 180 815 | 0 | 0.53 | 0 | 0–6.96 |
| UZ | 11 826 | 288 574 | 0 | 0.51 | 0 | 0–7.23 |
| Total | 23 790 | 581 657 | 0 | 1.37 | 0 | 0–2.69 |
| Male co-twins from unlike sex pairs (born 1926–67) | 13 820 | 337 176 | 0 | 0.82 | 0 | 0–4.50 |
| All Nordic twin cohorts | | | | | | |
| MZ | 14 460 | 370 110 | 2 | 3.49 | 0.58 | 0.07–2.07 |
| DZ | 41 358 | 1 034 596 | 5 | 7.02 | 0.71 | 0.23–1.66 |
| UZ | 16 255 | 380 735 | 2 | 1.31 | 1.53 | 0.19–5.52 |
| Total | 72 073 | 1 785 441 | 9 | 11.82 | 0.76 | 0.35–1.45 |
| US 1946–95 (born 1917–27) | | | | | | |
| MZ | 11 617 | 528 243 | 1 | 1.66 | 0.60 | 0.02–3.36 |
| DZ | 14 796 | 665 922 | 1 | 2.09 | 0.48 | 0.01–2.67 |
| UZ | 4507 | 194 781 | 0 | 0.59 | 0 | 0–6.25 |
| Total | 30 920 | 1 388 946 | 2 | 4.34 | 0.46 | 0.06–1.66 |
| All twin cohorts | | | | | | |
| MZ | 26 077 | 898 353 | 3 | 5.15 | 0.58 | 0.12–1.70 |
| DZ | 56 154 | 1 756 672 | 6 | 9.11 | 0.66 | 0.24–1.43 |
| UZ | 33 004 | 874 500 | 2 | 1.90 | 1.05 | 0.13–3.80 |
| Total | 115 235 | 3 529 525 | 11 | 16.16 | 0.68 | 0.34–1.22 |

[a]Person-years at risk. [b]Number of observed cases of MBC. [c]Expected number of cases of MBC based on age- and calendar-year incidence rates for each country. [d]Standardized rate ratio (incidence for Nordic cohorts, mortality for US cohort). [e]95% confidence interval. [f]MZ, monozygous twins; DZ, dizygous twins; UZ, twins of unknown zygosity.

either by the corresponding cancer incidence in the appropriate Nordic country or by death rate of US white male population. The standardized rate ratios (SRR) were computed by dividing the observed number of cases by the number expected; they denote standardized incidence ratios for the Nordic cohorts and standardized mortality ratios for the US cohort. The 95% confidence intervals (CI) were calculated from the Poisson distribution. We calculated the Breslow–Day statistic to test for heterogeneity of the SRR across studies (Breslow and Day, 1987).

Overall, we observed 11 cases of MBC versus 16.16 expected cases (Table 1). We found no evidence that male twins were at increased risk for MBC when compared with the general population (SRR 0.68, 95% CI 0.34–1.22). There was no evidence of heterogeneity of the SRRs across studies ($\chi^2 = 3.66$, $P = 0.30$), and restricting the analysis to incident cases of MBC from the similar Nordic cohorts made no substantive difference to the SRR (9 cases observed, 11.82 expected, SRR 0.76, 95% CI 0.35–1.45).

The occurrence of MBC appeared similarly reduced among MZ (SRR 0.58, 95% CI 0.12–1.70) and DZ twins (SRR 0.66, 95% CI 0.24–1.43) compared with population expectation, while for male twins of unknown zygosity, the SRR showed a marginal, non-significant increase (1.05, 95% CI 0.13–3.80). There were no cases of MBC observed among the Swedish cohort of 13 820 male co-twins from unlike-sex pairs during 337 176 person-years at risk (0.82 expected, SRR 0, 95% CI 0–4.50).

We have found no support for the notion that male offspring of twin pregnancies are at higher risk for breast cancer than the general population. Our findings agree with a recent meta-analysis of breast cancer in female twins, which found a pooled standardized incidence ratio of 1.05 (95% CI 0.99–1.1) and thus excluded any important increase in breast cancer risk among female twins of all ages (Verkasalo et al, 1999b). The meta-analysis was based on 1323 cases of female breast cancer diagnosed among three Nordic twin cohorts. On the other hand, a recent US study reported a relative risk of 1.72 (95% CI 1.22–2.42) for breast cancer among postmenopausal female twins based upon 35 cases of breast cancer (5 cases among MZ, 23 among DZ and 7 among twins of unknown zygosity) (Cerhan et al, 2000).

Our record-linkage study approach is the most efficient epidemiological design to test our hypothesis. Nevertheless, even though we pooled data from three national twin registers and a further large population-based register, our study had limited power to provide precise risk estimates. This limitation will apply to all studies attempting to investigate the ‘in utero exposure

 *© 2000 Cancer Research Campaign*

hypothesis' in MBC, due to the rarity of both the exposure and the outcome. In particular, our estimate of the risk of MBC among male co-twins of unlike-sex pairs lacks precision since only one registry had assembled a cohort of this type. While other twin cohorts certainly exist (Boomsma, 1998), very few combine the characteristics of large sample size and long duration of follow-up necessary to generate cases of this rare malignancy. Moreover, to our knowledge, no other twin cohorts besides those involved in this pooled analysis have directly assessed the occurrence of breast cancer among male twins.

A frequent limitation of record-linkage methods is the inability to adjust for potentially confounding factors other than age. However, few risk factors are known for MBC and the population prevalence of the commonly cited risk factors, such as Klinefelter's syndrome or exposure to ionizing radiation (Thomas, 1993), is very low and unlikely to account for the null findings of the present study.

We conclude that if in utero exposure to high levels of oestrogen is involved in the development of male breast cancer, then the influence is likely to be very small.

### ACKNOWLEDGEMENTS

We thank Hannu Kiviranta, Mariedal Konsult AB for the computer work on the Swedish data set and Eero Pukkala, Finnish Cancer Registry, for calculating the standardized incidence ratios of the Finnish data set. The Danish cancer twin study was supported by research grants 36/79 from The Danish Cancer Society and R35 CA 42581 and PO1-AG08761 from the United States National Cancer Institute and National Institute on Aging research, respectively. The Finnish twin study was supported by the Academy of Finland and the Finnish Cancer Foundations. The Swedish Twin Registry is supported by grants from the Swedish Council for the Planning and Coordination of Research (FRN), the Swedish Social Research Council, and the MacArthur Foundation Research Network on Successful Aging. Work on the American twins was funded by Department of Veterans Affairs contract V101(93)P-1637, TO#9. David Whiteman was funded by a Nuffield Medical Research Fellowship from the University of Oxford.

The opinions and assertions contained herein are those of the authors and are not to be construed as reflecting the views or position of the National Academy of Sciences, the Institute of Medicine, or the National Research Council.

### REFERENCES

Boomsma DI (1998) Twin registers in Europe: an overview. *Twin Research* **1**: 34–51

Breslow NE and Day NE (1987) Statistical methods in cancer research. Volume II – The design and analysis of cohort studies. IARC Scientific Publications No 82. International Agency for Research on Cancer: Lyon

Cederlof R, Friberg L and Lundman T (1977) The interaction of smoking, environment and heredity and their implications for disease etiology: a report of epidemiological studies on the Swedish Twin Registries. *Acta Med Scand* **612**: 1–128

Cerhan JR, Kushi LH, Olson JE, Rich SS, Zheng W, Folsom AR and Sellers TA (2000) Twinship and risk of postmenopausal breast cancer. *J Natl Cancer Inst* **92**: 261–265

Friedman LS, Gayther SA, Kurosaki T, Gordon D, Noble B, Casey G, Ponder BAJ and Anton-Cluver H (1997) Mutation analysis of BRCA1 and BRCA2 in a male breast cancer population. *Am J Hum Genet* **60**: 313–319

Hauge M (1981) The Danish Twin Register. In: Mednick SA, Baert AE, Bachmann BP (eds) *Prospective Longitudinal Research*. Oxford Medical Publications: Oxford, pp. 217–222

Jablon S, Neel JV, Gershowitz H and Atkinson GF (1967) The NAS-NRC Twin Panel: methods of construction, zygosity diagnosis and proposed use. *Am J Hum Genet* **19**: 133–161

Lichtenstein P, Holm N, Verkasalo PK, Iliadou A, Kaprio J, Koskenvuo M, Pukkala E, Skytthe A and Hemminki K (2000) Environmental and heritable factors in the causation of cancer. *New Engl J Med* **343**: 78–85

London WP (1993) Male sexual development in "a sea of oestrogen". *Lancet* **342**: 124

Mavraki E, Gray IC, Bishop DT and Spurr NK (1997) Germline BRCA2 mutations in men with breast cancer. *Br J Cancer* **76**: 1428–1431

Medlund P, Cederlöf R, Floderus-Myrhed B, Friberg L and Sörensen S (1976) A new Swedish Twin Registry: containing environmental and medical base line data from about 14 000 same-sexed pairs born 1926–1958. *Acta Med Scand* **600**: 1–111.

Sasco AJ, Lowenfels AB and Pasker-De Jong P (1993) Review article: Epidemiology of male breast cancer. A meta-analysis of published case-control studies and discussion of selected aetiological factors. *Int J Cancer* **53**: 538–549

TambyRaja RL and Ratnam SS (1981) Plasma steroid changes in twin pregnancies. In: Gedda I, Parisi P, Nance W (eds) *Twin Research 3. Progress in Clinical and Biological Research*, volume 69A. Alan R. Liss: New York, p 189–195

Thomas DB (1993) Breast cancer in men. *Epidemiol Rev* **15**: 220–231

Thomas HV, Murphy MF, Key TJ, Fentiman IS, Allen DS and Kinlen LJ (1998) Pregnancy and menstrual hormone levels in mothers of twins compared to mothers of singletons. *Ann Hum Biol* **25**: 69–75

Trichopoulos D (1990) Hypothesis: does breast cancer originate in utero? *Lancet* **335**: 939–940

Verkasalo PK, Kaprio J, Koskenvuo M and Pukkala E (1999a) Genetic predisposition, environment and cancer incidence: a nationwide twin study in Finland. *Int J Cancer* **83**: 743–749.

Verkasalo PK, Kaprio J, Pukkala E and Koskenvuo M (1999b) Breast cancer risk in monozygotic and dizygotic female twins: a 20-year population-based cohort study in Finland from 1976 to 1995. *Cancer Epidemiol Biomarkers Prev* **8**: 271–274

© 2000 Cancer Research Campaign

